# Emergency Department-initiated High-flow Nasal Cannula for COVID-19 Respiratory Distress

**DOI:** 10.5811/westjem.2021.3.50116

**Published:** 2021-07-20

**Authors:** Zachary J. Jarou, David G. Beiser, Willard W. Sharp, Ravi Chacko, Deirdre Goode, Daniel S. Rubin, Dinesh Kurian, Allison Dalton, Stephen R. Estime, Michael O’Connor, Bhakti K. Patel, John P. Kress, Thomas F. Spiegel

**Affiliations:** * University of Chicago, Section of Emergency Medicine, Department of Medicine, Chicago, Illinois; † University of Chicago, Department of Anesthesia and Critical Care, Chicago, Illinois; ‡ University of Chicago, Section of Pulmonary and Critical Care Medicine, Department of Medicine, Chicago, Illinois

## Abstract

**Introduction:**

Patients with coronavirus disease 2019 (COVID-19) can develop rapidly progressive respiratory failure. Ventilation strategies during the COVID-19 pandemic seek to minimize patient mortality. In this study we examine associations between the availability of emergency department (ED)-initiated high-flow nasal cannula (HFNC) for patients presenting with COVID-19 respiratory distress and outcomes, including rates of endotracheal intubation (ETT), mortality, and hospital length of stay.

**Methods:**

We performed a retrospective, non-concurrent cohort study of patients with COVID-19 respiratory distress presenting to the ED who required HFNC or ETT in the ED or within 24 hours following ED departure. Comparisons were made between patients presenting before and after the introduction of an ED-HFNC protocol.

**Results:**

Use of HFNC was associated with a reduced rate of ETT in the ED (46.4% vs 26.3%, P <0.001) and decreased the cumulative proportion of patients who required ETT within 24 hours of ED departure (85.7% vs 32.6%, P <0.001) or during their entire hospitalization (89.3% vs 48.4%, P <0.001). Using HFNC was also associated with a trend toward increased survival to hospital discharge; however, this was not statistically significant (50.0% vs 68.4%, P = 0.115). There was no impact on intensive care unit or hospital length of stay. Demographics, comorbidities, and illness severity were similar in both cohorts.

**Conclusions:**

The institution of an ED-HFNC protocol for patients with COVID-19 respiratory distress was associated with reductions in the rate of ETT. Early initiation of HFNC is a promising strategy for avoiding ETT and improving outcomes in patients with COVID-19.

## INTRODUCTION

### Background

Global healthcare resources have been tested by the rapid spread of severe acute respiratory syndrome coronavirus 2 (SARS-CoV-2) leading to increased prevalence of the infectious syndrome known as coronavirus disease 2019 (COVID-19). Patients with COVID-19 can develop rapidly progressive respiratory failure over a period of hours to days.^1^ Early reports from Wuhan, China, suggested that early endotracheal intubation (ETT) was crucial for treating respiratory failure in patients with COVID-19 pneumonitis.^2^

Contemporary concerns regarding the risk of bio-aerosol dispersion during the use of non-invasive ventilation methods led to hospital policies and approaches that favored ETT with closed ventilatory circuits and viral filters over non-invasive ventilation to limit infectious spread to medical professionals.^3^–^7^

### Importance

Ventilation strategies during the COVID-19 pandemic seek to minimize patient mortality while also reducing infectious risk to medical professionals. With limited supplies of ventilators, negative pressure rooms and personal protective equipment (PPE), the rapid spread of COVID-19 within communities experiencing severe outbreaks can quickly overwhelm hospital resources.^8^ Prior to COVID-19, it was known that high-flow nasal cannula (HFNC) may decrease the need for ETT in patients with acute hypoxemic respiratory failure without increasing mortality. 9 And HFNC has shown promising results for reducing ETT in patients with other severe respiratory viruses such as H1N1.^10^ Developing a better understanding of the impact of HFNC on patient outcomes and healthcare worker safety is critical.

### Goals of This Investigation

At the beginning of the COVID-19 pandemic, our institution restricted the use of HFNC in the ED for COVID-19 patients; however, after noting improved outcomes in patients receiving HFNC in our medical intensive care unit (ICU), our ED instituted the use of HFNC in select negative pressure rooms. The timeline of institutional policies supporting early ETT of COVID-positive patients in the ED and subsequent implementation of ED-initiated HFNC provided a natural before-and-after experiment of two patient cohorts whose outcomes could be studied.

The objective of this retrospective cohort study was to determine the potential impact of ED-initiated HFNC for the treatment of COVID-19 respiratory failure by looking at patient outcomes before and after its availability. We hypothesized that the availability of HFNC in the ED would be associated with a decreased proportion of patients intubated in the ED, decreased proportion of patients intubated within the first 24 hours of hospitalization, decreased hospital and ICU length of stay, and improved survival.

### Methods

#### Study Design and Setting

This retrospective, non-concurrent cohort study was approved by the University of Chicago Institutional Review Board (IRB20-0781) and conducted at the University of Chicago Medical Center, a large, urban, quaternary, academic medical center and Level I trauma center. According to the hospital’s 2018–2019 Community Health Needs Assessment, the population of the 12 ZIP code service area is 625,707, and is 76.7% non-Hispanic Black/African American, 12.3% Hispanic/Latino, and 7.8% non-Hispanic. Annual ED volume was 108,188 as of June 2020, 68.9% of which were adult visits. On January 24, 2020, the University of Chicago Hospital Incident Command System (HICS) was activated and travel-screening for COVID-19 was initiated. On March 18, 2020, in response to international reports of healthcare worker SARS-CoV-2 transmission following aerosolizing procedures, HICS restricted the use of all aerosol-generating procedures including nebulizers and non-invasive positive pressure ventilation (NIPPV) in all non-ICU settings, including the ED. All patients requiring greater than six liters per minute of supplemental oxygen by nasal cannula, those with severe respiratory fatigue, hypercarbia, or those unable to protect their airways received ETT in the ED.

On April 6, institutional policies changed to allow for the use of high-flow nasal cannula (HFNC) in the ED for COVID-positive patients requiring greater than six liters of oxygen per minute by nasal cannula. Patients receiving HFNC were required to be placed in a negative pressure room with an anteroom to limit the spread of aerosolized virus. The HFNC was initiated at a flow rate of 40 liters per minute and 100% fraction of inspired oxygen (FiO_2_). The flow rate was titrated up to 60 liters per minute as needed to decrease work of breathing and maintain a respiratory rate of less than 30 breaths per minute. FiO_2_ was titrated to maintain an oxygen saturation between 92–96%. Decisions about which patients needed ETT rather than HFNC prior to ED departure were made by the bedside emergency physician. Some patients were transiently placed on HFNC while in the ED but were able to be de-escalated to nasal cannula prior to ED departure. Results of this study are reported in accordance with the STROBE Guidelines (Strengthening the Reporting of Observational Studies in Epidemiology).^11^

#### Selection of Participants

We included in the study all patients greater than or equal to 18 years old who screened positive for COVID-19, were admitted to the hospital from the University of Chicago adult ED between March 1–May 22, 2020, and required HFNC or ETT within the first 24 hours of hospitalization. Exclusion criteria included patients who were discharged from the ED, sent directly to labor and delivery, expired in the ED, had an operative procedure during their admission, or patients who were transiently placed on HFNC in the ED but de-escalated to nasal cannula prior to ED departure. COVID-19 infections were confirmed using Roche cobas (Roche Diagnostics Corporation, Indianapolis, IN) or Cepheid Xpert Xpress (Cepheid, Sunnyvale, CA) SARS-CoV-2 qualitative reverse transcriptase polymerase chain reaction assays. Testing may have occurred at an outpatient clinic or curbside locations prior to visiting the ED, in the ED, or after admission. Patients were considered positive during their hospital encounter if they had a positive result within 14 days prior to ED arrival or prior to hospital discharge. The study was approved by the University of Chicago Institutional Review Board (IRB20-0781), and the need for informed consent was waived as all patient data were obtained through a de-identified data mart.

#### Measurements

All data provided for this study were obtained through a de-identified COVID-19 data mart created and maintained by the University of Chicago Center for Research Informatics (CRI). The CRI data mart comprised multiple tables, including the following: patient demographic information; admit/discharge/transfer (ADT) events, encounters, flowsheets, diagnosis and problem lists; smoking history; lab values; inpatient diagnosis-related groups (DRG); de-identified notes; and medication administrations.

Patient ages were calculated for each encounter using the number of years between patient birth date and the ADT timestamp of ED arrival. The timing of respiratory interventions was determined by grouping respiratory flowsheet events by patient ID and oxygen delivery method. The earliest timestamps for HFNC and intubation during each encounter were saved for each patient where applicable. Patient comorbidities were determined using *International Classification of Diseases, 10th Revision* (ICD-10) codes from their diagnosis and problem lists. We mapped ICD-10 codes using methods previously described by Charlson, Elixhauser, and van Walraven.^12^–^14^ Hypertension, diabetes, chronic obstructive pulmonary disease (COPD), and chronic kidney disease were codified according to Elixhauser. Acute myocardial infarctions were codified according to Charlson. Total weighted Charlson and van Walraven-weighted Elixhauser scores were also reported. We determined survival at hospital discharge using a status within the patient demographics table provided by CRI. This status was compared and corrected using death notes and hospital discharge disposition status for patients in our cohorts.

To assess for potential confounding bias due to patient-level differences in the composition of each cohort, we compared the cohorts to one another regarding patient age, gender, race, ethnicity, comorbidities, ED vital signs, illness severity, and lab values/biomarkers. Comorbidities controlled for included those previously associated with increased mortality in COVID-19 (hypertension, diabetes mellitus, coronary artery disease, COPD, chronic kidney disease, anemia), as well as those that comprise the Charlson and Elixhauser scoring systems, which have strong prior validity evidence to predict inpatient mortality for both COVID and non-COVID patients.^11^–^15^ We compared illness severity using each patient’s mean arterial oxygen partial pressure/fractional inspired ratio (PaO_2_/FiO_2_) ratio within the first 24 hours of hospitalization, as well as initial sequential organ failure assessment (SOFA) score upon arrival to the ICU. The labs/biomarkers that were selected to ensure similarities between patient cohorts are those that have been previously associated with increased mortality, including complete blood counts, serum bicarbonate, blood urea nitrogen, serum creatinine, glucose, alanine aminotransferase (ALT), lactate dehydrogenase, creatinine kinase, troponin, prothrombin time, D-dimer, ferritin, interleukin-6 and C-reactive protein.^16^–^18^ We also attempted to control for confounding by comparing differences in the rate of in-patient treatment with remdesivir, which has previously been shown to decrease hospital length of stay.^19^

Additional details regarding variable transformation are available in the Supplemental Methods.

The primary outcome variables were the maximum levels of respiratory support at ED departure, within the first 24 hours after ED departure, and through the entire duration of hospitalization, as well as survival at hospital discharge. Secondary outcome variables included total inpatient and ICU lengths of stay.

#### Data Analysis

We performed an *a priori* sample size calculation to detect a 50% decrease in the proportion of patients requiring ETT within 24 hours of hospitalization, from 90% prior to the availability of ED HFNC to 45% following the availability of ED HFNC, resulting in a minimum sample size of 42 patients, using alpha of 0.05 and a power of 90% (G*Power v3.1; Faul, Erdfelder, Buchner, & Lang [2009]). The decision to power our study to detect a 50% reduction in ETT was based upon our personal experiences in caring for patients during the time periods prior to and following the availability of ED HFNC. We performed all data extraction, transformation, and analysis using RStudio version 1.2.5001 running R version 3.5.1 and *tidyverse* 1.2.1 (RStudio, PBC, Boston, MA). We mapped ICD-10 codes for each patient to individual comorbidities using the *comorbidity* package.^20^ The distribution of all variables for each cohort was visualized using the *explore* package,^21^ and summary statistics were calculated using the *arsenal* package.^22^ All missing values were imputed using *missForest*, a non-parametric, random forest-based method.^39^ As the visualizations of the distributions of our continuous variables displayed that they were not normally distributed, continuous variables were reported using medians and interquartile ranges (IQR). Categorical variables were described using frequency and percentages. We compared continuous variables using the Wilcoxon rank-sum test and categorical variables using Fisher’s exact test. *P*-values less than 0.05 were considered to be statistically significant.

## RESULTS

### Characteristics of Study Subjects

**Figure 1:**
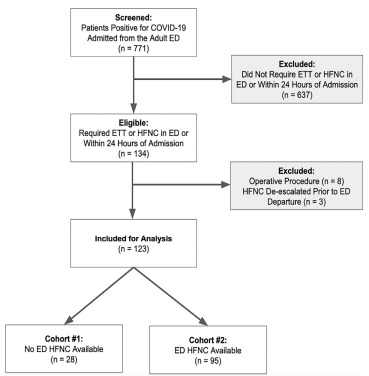
Flow chart of patient screening, eligibility, inclusion, exclusion. *ED*, emergency department; *ETT*, endotracheal intubation; *HFNC*, high-flow nasal cannula.

There were 771 encounters with COVID-19-positive patients greater than or equal to 18 years old seen in the adult ED resulting in hospital admission. A total of 134 patients required HFNC or ETT within 24 hours of admission. We excluded eight patients who underwent operative procedures during hospitalization and three patients who were started on ED-HFNC but de-escalated to nasal cannula prior to ED departure. Of the 123 patients meeting both the inclusion and exclusion criteria, 28 were seen prior to the availability of ED-HFNC and 95 were seen following the availability of ED-HFNC. See Figure 1 for a flow diagram of patient screening, eligibility, inclusion, and exclusion.

The median age of the study population was 65 years (IQR 57–75). Patients were predominantly Black/African-American (85.4%) and non-Hispanic (90.2%). Participants were 52.0% male. There were no statistically significant differences between the demographics of each group.

The median body mass index was 31.4 (IQR 25.2–38.5], and there were no differences in smoking status or the prevalence of comorbidities between the two groups (48.8% diabetes, 83.7% hypertension, 44.7% chronic kidney disease, 27.6% COPD, 22.8% myocardial infarction). The median weighted Charlson score was 4 (IQR 2–6), and the median van Walraven (Elixhauser) score was 17 (IQR 9.0–26.5). There were no differences in Charlson or van Walraven scores or any of their component comorbidities between the two groups.

When comparing the worst ED vital signs for each patient, as defined by the maximum recorded heart rate, temperature, and respiratory rate, and minimum recorded systolic blood pressure and oxygen saturation, we found no statistically significant differences between the two cohorts. Similarly, there were no statistically significant differences between the two cohorts in terms of illness severity, as defined by the median PaO2/FiO2 ratio during the first 24 hours of hospitalization and the SOFA score upon ICU admission. There were no differences in lab values between the two groups. Overall, 34.1% of patients received remdesivir after admission. There was no statistical difference in the rate of treatment with remdesivir between the two groups. Table 1 shows some characteristics between the two groups. Please see Supplemental Table for complete information on the demographics, comorbidities, vital signs, and laboratory values between the two groups.

### Main Results

For patients with COVID-19 respiratory distress requiring ETT/HFNC within the first 24 hours of hospitalization, the introduction of ED-initiated HFNC was associated with a reduced rate of ETT in the ED (46.4% vs 26.3%, *P* <0.001). The availability of ED-HFNC was also associated with a significant decrease in the cumulative proportion of patients who required ETT within 24 hours of hospitalization (85.7% vs 32.6%, *P* <0.001) and throughout their entire admission (89.3% vs 48.4%, *P* <0.001).

While there were trends toward increased survival (50.0% vs 68.4%) and decreased ICU length of stay (median 8.6 days [IQR 5.1–10.9] vs. 6.0 days [IQR 2.9–13.5]), these findings were not statistically significant. There was no difference in the median total inpatient length of stay between the two study periods. See Table 2 for complete information comparing the primary and secondary outcomes between patient cohorts.

## DISCUSSION

Overall, our study suggests that making HFNC available as a treatment option in the ED for patients experiencing respiratory distress due to COVID-19 was associated with a significantly reduced rate of ETT in the ED and reduced intubation through the entire period of hospitalization. While there were trends toward improved survival and decreased ICU length of stay, these findings were not statistically significant.

A prior case series evaluating the use of HFNC for patients with severe H1N1 influenza pneumonitis found that 45% of patients receiving HFNC (9/20) never required intubation, suggesting that HFNC may play a role in the treatment of infectious severe hypoxemic respiratory failure.^10^ For COVID-19-associated respiratory failure, Jiangsu Province in China reported better survival outcomes than Hubei Province (3.33% vs. 4.34%), which they attributed to early recognition of high-risk and critically ill patients to allow early intervention with a multi-pronged approach that included HFNC or NIPPV, along with fluid restriction and early proning.^23^ This approach was associated with <1% of Jiangsu Province patients requiring ETT compared to the national average of 2.3%.^24^

While the results of this study support the use of ED-initiated HFNC for COVID-19-associated respiratory distress, there are some risks and limitations of HFNC that must be considered. Given the potential for aerosolization of the SARS-CoV-2 virus,^25^ we recommend that HFNC be used only in single-occupancy, negative pressure airborne isolation rooms that are entered by a limited number of care team members who are appropriately trained in the proper donning and doffing of personal protective equipment.^26^ To facilitate the safe use of HFNC, our hospital constructed negative anteroom chambers for some of our existing negative pressure rooms. Also, not all patients are suitable candidates for HFNC; these include patients who are unable to protect their airways, need operative procedures, or with severe acidosis or hypercarbia, and those who have continued respiratory distress despite being treated with HFNC. Furthermore, there may be risks associated with the overuse of HFNC and some pre-COVID-19 reports have suggested that failure of HFNC may delay intubation and increase mortality.^27^ The “ROX index,” calculated as the ratio of oxygen saturation to FiO_2_, has recently been developed to help predict which patients will succeed with HFNC or progress to needing ETT; 28,29 however, this was not part of our institutional protocol.

Some studies have shown that HFNC causes minimal bio-aerosol dispersion,^3^–^4^ while others have shown that HFNC increased droplet dispersion to levels that are unacceptable according to World Health Organization guidelines.^5^ Early recommendations favored ETT over HFNC as ETT creates a closed circuit with high efficiency particulate air or viral filters that limit infectious spread to medical professionals.^6^ It was recommended that patients not be placed on HFNC until viral clearance had been proven.^7^ Compared to NIPVV, HFNC has been shown to generate fewer aerosols.^30^ Nurses treating patients with SARS-CoV-1 were also found to be at higher risk for developing SARS when patients were being treated with NIPPV.^31^

Although not formally included as part of our study, we did not see an increased rate of COVID-19 among healthcare workers as a result of treating COVID-positive patients with HFNC. During the period before ED-HFNC two physicians and four nurses working in our ED tested positive for SARS-CoV-2; in the time period following ED-HFNC, no physicians and six nurses tested positive, none of whom were found to have provided direct patient care to any COVID-19-positive patient on HFNC.

We hypothesize that the two primary mechanisms by which HFNC might improve patient outcomes include the following: 1) earlier respiratory support for patients who need it; and 2) decreased complications associated with ETT. When the only available option to emergency physicians is ETT or no ETT, we observed that nearly half of all patients who ultimately required ETT/HFNC within 24 hours of ED departure did not have these interventions in the ED. This finding indicates that there may have been an opportunity to provide earlier respiratory support and prevent later decompensation, a trajectory that may have ultimately impacted survival. The widely known FLORALI trial showed that HFNC did not reduce the risk of intubation in patients with acute hypoxemic respiratory failure but was associated with improved 90-day mortality^32^; however, a more recent meta-analysis has shown the opposite—that HFNC reduces the need for intubation with no reductions in mortality or hospital or ICU length of stay.^9^

The risks of ETT are numerous, including increased risk of ventilator-associated infections, barotrauma, extended ICU stays, and adverse reactions to sedation.^33^ Furthermore, concerns about patient self-induced lung injury (P-SILI) that have been cited in earlier viewpoints favoring early intubation have been called into question. The idea that patients with heightened respiratory drive have maladaptively high tidal volumes that then induce more severe acute respiratory distress syndrome is based on only two studies, each of which has significant limitations.^34^

While these data provide compelling support for the use of ED-HFNC in the treatment of COVID-19 pneumonitis, it will be important to consider which patients are at high risk of HFNC-failure as determined by their ROX index, as well as other treatments that could be initiated in the ED that could augment patient outcomes. A recent study of early self-proning in awake, non-intubated, COVID-19-positive patients in the ED found significant improvements in oxygen saturation within five minutes,^35^ and a randomized controlled trial comparing early prone positioning with HFNC vs HFNC alone is currently underway.^36^ It may also be worth further studying ways to use HFNC in austere settings such as temporary alternative care locations or in EDs operating beyond capacity where individual treatment rooms are not available, along with a more protocolized approach to measuring the risk of transmission to healthcare workers.

## LIMITATIONS

This study has several limitations. As part of a retrospective cohort study, patients were not randomized with respect to which interventions they received and thus causation could not be established. We also recognize the risk of chronology bias in studying non-concurrent cohorts during a pandemic where practice is likely to quickly evolve in response to emerging literature in ways that were not captured by our analyses. Such unmeasured changes in practice would likely have the most impact upon distal outcomes such as hospital discharge. The sample size for this study was calculated to detect a 50% reduction in the rate of ETT; therefore, it was underpowered to detect differences in mortality rates associated with ED-HFNC. Also, as a study of a single, urban, academic medical center with a predominately African-American/Black patient population, our results may not be entirely generalizable, although given the increased incidence of COVID-19 in Black communities, these results may be of particular importance for this population.

While it may seem surprising that patients requiring HFNC or ETT did not have higher temperatures, this may be related to the use of infrared forehead thermometers, which have previously been shown not to be as accurate as other measurement methods.^,^ Additionally, analysis was performed using de-identified information contained within a data mart rather than having the ability to review individual patient charts directly in the electronic health record, which limited the ability to control for certain potential confounders, such as prone positioning and traditional or radiographic-based pneumonia severity scores, since these were not included in the data mart.

## CONCLUSION

Given our findings, we believe that despite early recommendations against its use, high-flow nasal cannula is a treatment option that should be considered for patients with COVID-19. We encourage hospital systems and emergency departments to closely evaluate their internal resources and consider deploying HFNC as a front-line treatment for patients with suspected or confirmed COVID-19 presenting with respiratory distress.

**Population Health Research Capsule**

What do we already know about this issue?

*Patients diagnosed with coronavirus disease 2019 (COVID-19) frequently develop severe respiratory distress requiring significant ventilatory support*.

What was the research question?

*Does the availability of ED-initiated high flow nasal cannula (HFNC) reduce the rate of endotracheal intubation (ETT) for patients with COVID-related respiratory distress?*

What was the major finding of the study?

*For patients with severe COVID, the availability of ED-HFNC reduced the rate of ETT in the ED, within the first 24 hours of hospitalization, and throughout their entire hospitalization*.

How does this improve population health?

*The use of ED-HFNC reduces the need for ETT, allowing efficient allocation of ventilators, which may be a scarce resource, while also reducing exposure to ventilator-associated complications*.

## Supplementary Information





## Figures and Tables

**Table 1:** Characteristics COVID-19-positive patients seen in the emergency department (ED) before and after the availability of high-flow nasal cannula in the ED.

	1: No ED HFNC Available (n = 28)	2: ED HFNC Available (n = 95)	Total (n = 123)	P-value
Demographics				
Age				0.849
Median	69.0	65.0	65.0	
Q1, Q3	57.8, 73.0	57.0, 76.0	57.0, 75.0	
Gender				0.668
Male	16 (57.1%)	48 (50.5%)	64 (52.0%)	
Female	12 (42.9%)	47 (49.5%)	59 (48.0%)	
Race				0.642
Black/African-American	25 (89.3%)	80 (84.2%)	105 (85.4%)	
White	2 (7.1%)	6 (6.3%)	8 (6.5%)	
More than one race	0 (0.0%)	6 (6.3%)	6 (4.9%)	
Other/unknown	1 (3.6%)	3 (3.2%)	4 (3.3%)	
Ethnicity				0.239
Not Hispanic or Latino	26 (92.9%)	85 (89.5%)	111 (90.2%)	
Hispanic or Latino	0 (0.0%)	7 (7.4%)	7 (5.7%)	
Unknown	2 (7.1%)	3 (3.2%)	5 (4.1%)	
Comorbidities				
Body mass index				0.263
Median	31.9	30.8	31.4	
Q1, Q3	29.8, 38.8	24.9, 38.0	25.2, 38.5	
Chronic kidney disease	12 (42.9%)	43 (45.3%)	55 (44.7%)	0.999
Chronic obstructive pulmonary disease	7 (25.0%)	27 (28.4%)	34 (27.6%)	0.813
Diabetes mellitus	15 (53.6%)	45 (47.4%)	60 (48.8%)	0.668
Hypertension	20 (71.4%)	83 (87.4%)	103 (83.7%)	0.07
Myocardial infarction	6 (21.4%)	22 (23.2%)	28 (22.8%)	0.999
Smoking status				0.058
Current Smoker	1 (3.6%)	6 (6.3%)	7 (5.7%)	
Former Smoker	13 (46.4%)	27 (28.4%)	40 (32.5%)	
Never Smoker	3 (10.7%)	32 (33.7%)	35 (28.5%)	
Unknown	11 (39.3%)	30 (31.6%)	41 (33.3%)	
Weighted Charlson score				0.989
Median	3.5	4	4	
Q1, Q3	1.8, 5.0	2.0, 6.0	2.0, 6.0	
Weighted Elixhauser score (Van Walraven)				0.959
Median	15	18	17	
Q1, Q3	8.2, 22.2	9.0, 27.5	9.0, 26.5	

* Full table included as a supplemental.

*ED*, emergency department; *HFNC*, high-flow nasal cannula.

**Table 2:** Patient outcomes before and sfter the availability of high-flow nasal cannula initiated in the emergency department.

	No ED HFNC Available (n = 28)	ED HFNC Available (n = 95)	Total (n = 123)	*P*-value
Primary outcomes				
Maximum respiratory support at ED departure				< 0.001
ETT	13 (46.4%)	25 (26.3%)	38 (30.9%)	
HFNC	0 (0.0%)	59 (62.1%)	59 (48.0%)	
No ETT/HFNC	15 (53.6%)	11 (11.6%)	26 (21.1%)	
Maximum respiratory support within 24 hours of hospitalization				< 0.001
ETT	24 (85.7%)	31 (32.6%)	55 (44.7%)	
HFNC	4 (14.3%)	64 (67.4%)	68 (55.3%)	
Maximum respiratory support during entire hospitalization				< 0.001
ETT	25 (89.3%)	46 (48.4%)	71 (57.7%)	
HFNC	3 (10.7%)	49 (51.6%)	52 (42.3%)	
Survival at hospital discharge				0.115
Alive	14 (50.0%)	65 (68.4%)	79 (64.2%)	
Deceased	14 (50.0%)	30 (31.6%)	44 (35.8%)	
Secondary outcomes				
Inpatient length of stay (days)				0.713
Median	9.9	10.1	10.0	
Q1, Q3	7.6, 18.5	6.9, 16.1	7.0, 16.7	
ICU length of stay (days)				0.305
Median	8.6	6.0	6.9	
Q1, Q3	5.1, 10.9	2.9, 13.5	3.0, 13.5	

*ED*, emergency department; *HFNC*, high-flow nasal cannula; *ETT*, endotracheal intubation; *ICU*, intensive care unit.
