# Supplementary material for: Emergency Department-initiated High-flow Nasal Cannula for COVID-19 Respiratory Distress
Source: West J Emerg Med. 2021 Jul 20;22(4):979–87. doi: 10.5811/westjem.2021.3.50116 (PMC8328178; doi:10.5811/westjem.2021.3.50116)
Supplement: Supplementary file 1 [file wjem-22-979-s001.docx]

**Supplemental Table.** Characteristics COVID-19-positive patients seen in the emergency department (ED) before and after the availability of high-flow nasal cannula in the ED.

|  | | **1: No ED HFNC Available**  **(n = 28)** | **2: ED HFNC Available**  **(n = 95)** | **Total**  **(n = 123)** | ***P*-value** |
| --- | --- | --- | --- | --- | --- |
| **Demographics** | | | | | |
| **Age** | |  |  |  | 0.849 |
|  | Median | 69.0 | 65.0 | 65.0 |  |
|  | Q1, Q3 | 57.8, 73.0 | 57.0, 76.0 | 57.0, 75.0 |  |
| **Gender** | |  |  |  | 0.668 |
|  | Male | 16 (57.1%) | 48 (50.5%) | 64 (52.0%) |  |
|  | Female | 12 (42.9%) | 47 (49.5%) | 59 (48.0%) |  |
| **Race** | |  |  |  | 0.642 |
|  | Black/African-American | 25 (89.3%) | 80 (84.2%) | 105 (85.4%) |  |
|  | White | 2 (7.1%) | 6 (6.3%) | 8 (6.5%) |  |
|  | More than one race | 0 (0.0%) | 6 (6.3%) | 6 (4.9%) |  |
|  | Other/unknown | 1 (3.6%) | 3 (3.2%) | 4 (3.3%) |  |
| **Ethnicity** | |  |  |  | 0.239 |
|  | Not Hispanic or Latino | 26 (92.9%) | 85 (89.5%) | 111 (90.2%) |  |
|  | Hispanic or Latino | 0 (0.0%) | 7 (7.4%) | 7 (5.7%) |  |
|  | Unknown | 2 (7.1%) | 3 (3.2%) | 5 (4.1%) |  |
| **Comorbidities** | | | | | |
| **Body mass index** | |  |  |  | 0.263 |
|  | Median | 31.9 | 30.8 | 31.4 |  |
|  | Q1, Q3 | 29.8, 38.8 | 24.9, 38.0 | 25.2, 38.5 |  |
| **Chronic kidney disease** | | 12 (42.9%) | 43 (45.3%) | 55 (44.7%) | 0.999 |
| **Chronic obstructive pulmonary disease** | | 7 (25.0%) | 27 (28.4%) | 34 (27.6%) | 0.813 |
| **Diabetes mellitus** | | 15 (53.6%) | 45 (47.4%) | 60 (48.8%) | 0.668 |
| **Hypertension** | | 20 (71.4%) | 83 (87.4%) | 103 (83.7%) | 0.07 |
| **Myocardial infarction** | | 6 (21.4%) | 22 (23.2%) | 28 (22.8%) | 0.999 |
| **Smoking status** | |  |  |  | 0.058 |
|  | Current Smoker | 1 (3.6%) | 6 (6.3%) | 7 (5.7%) |  |
|  | Former Smoker | 13 (46.4%) | 27 (28.4%) | 40 (32.5%) |  |
|  | Never Smoker | 3 (10.7%) | 32 (33.7%) | 35 (28.5%) |  |
|  | Unknown | 11 (39.3%) | 30 (31.6%) | 41 (33.3%) |  |
| **Weighted Charlson score** | |  |  |  | 0.989 |
|  | Median | 3.5 | 4 | 4 |  |
|  | Q1, Q3 | 1.8, 5.0 | 2.0, 6.0 | 2.0, 6.0 |  |
| **Weighted Elixhauser score (Van Walraven)** | | | |  | 0.959 |
|  | Median | 15 | 18 | 17 |  |
|  | Q1, Q3 | 8.2, 22.2 | 9.0, 27.5 | 9.0, 26.5 |  |
| **ED Vital Signs** | | | | | |
| **Highest Heart Rate** | | | | | 0.229 |
|  | Median | 112 | 109 | 111 |  |
|  | Q1, Q3 | 103.8, 128.5 | 99.5, 122.5 | 100.5, 125.0 |  |
| **Lowest Pulse Oximetry (SpO2)** | | | | | 0.969 |
|  | Median | 85 | 85 | 85 |  |
|  | Q1, Q3 | 76.8, 87.0 | 74.0, 90.0 | 75.0, 89.5 |  |
| **Lowest Systolic Blood Pressure** | | | | | 0.415 |
|  | Median | 98 | 99 | 98 |  |
|  | Q1, Q3 | 84.8, 104.2 | 85.0, 113.0 | 84.5, 108.5 |  |
| **Highest Temperature (F)** | | | | | 0.078 |
|  | Median | 99.5 | 98.8 | 99.1 |  |
|  | Q1, Q3 | 98.6, 100.8 | 98.0, 100.0 | 98.1, 100.2 |  |
| **Highest Respiratory Rate** | | | | | 0.156 |
|  | Median | 32 | 34 | 33 |  |
|  | Q1, Q3 | 29.0, 35.8 | 30.0, 40.0 | 30.0, 39.0 |  |
| **Illness Severity** | | | | | |
| **PaO2/FiO2 Ratio During First 24 Hours of Hospitalization** | | | | | 0.284 |
|  | Median | 159.1 | 139.1 | 150.4 |  |
|  | Q1, Q3 | 115.5, 217.3 | 101.5, 199.5 | 101.5, 205.2 |  |
| **SOFA Score Upon ICU Arrival** | | | | | 0.065 |
|  | Median | 5 | 4 | 4 |  |
|  | Q1, Q3 | 3.8, 6.0 | 2.0, 6.0 | 2.0, 6.0 |  |
| **Labs - Complete Blood Count** | | | | | |
| **White Blood Cell Count** | |  |  |  | 0.511 |
|  | Median | 8.4 | 9.7 | 9.1 |  |
|  | Q1, Q3 | 7.6, 11.1 | 6.7, 14.1 | 6.8, 13.2 |  |
| **Absolute Lymphocytes** | |  |  |  | 0.761 |
|  | Median | 1.2 | 1 | 1.1 |  |
|  | Q1, Q3 | 0.7, 1.4 | 0.7, 1.6 | 0.7, 1.5 |  |
| **Absolute Neutrophils** | |  |  |  | 0.336 |
|  | Median | 6.2 | 7.5 | 7.1 |  |
|  | Q1, Q3 | 5.8, 7.5 | 5.0, 11.5 | 5.1, 10.7 |  |
| **Hemoglobin** | |  |  |  | 0.204 |
|  | Median | 13.2 | 12.8 | 12.9 |  |
|  | Q1, Q3 | 12.2, 14.9 | 11.1, 14.6 | 11.3, 14.7 |  |
| **RBC Distribution Width** | |  |  |  | 0.478 |
|  | Median | 14.4 | 14.6 | 14.6 |  |
|  | Q1, Q3 | 13.1, 16.4 | 13.5, 15.6 | 13.4, 15.8 |  |
| **Hematocrit** | |  |  |  | 0.302 |
|  | Median | 42.9 | 41.3 | 41.4 |  |
|  | Q1, Q3 | 38.9, 45.3 | 35.4, 45.5 | 36.5, 45.5 |  |
| **Platelet Count** | |  |  |  | 0.644 |
|  | Median | 214.5 | 212 | 212 |  |
|  | Q1, Q3 | 166.8, 255.2 | 158.5, 295.5 | 158.5, 286.0 |  |
| **Labs - Comprehensive Metabolic Panel** | | | | | |
| **Bicarbonate** | |  |  |  | 0.271 |
|  | Median | 21.5 | 20 | 20 |  |
|  | Q1, Q3 | 18.8, 24.0 | 17.0, 23.0 | 17.5, 23.0 |  |
| **Blood urea nitrogen** | |  |  |  | 0.24 |
|  | Median | 21.5 | 20 | 20 |  |
|  | Q1, Q3 | 18.8, 24.0 | 17.0, 23.0 | 17.5, 23.0 |  |
| **Creatinine** | |  |  |  | 0.84 |
|  | Median | 21.5 | 20 | 20 |  |
|  | Q1, Q3 | 18.8, 24.0 | 17.0, 23.0 | 17.5, 23.0 |  |
| **Glucose** | |  |  |  | 0.433 |
|  | Median | 21.5 | 20 | 20 |  |
|  | Q1, Q3 | 18.8, 24.0 | 17.0, 23.0 | 17.5, 23.0 |  |
| **Alanine aminotransferase** | |  |  |  | 0.809 |
|  | Median | 31 | 38 | 38 |  |
|  | Q1, Q3 | 19.8, 59.5 | 20.0, 56.0 | 20.0, 57.5 |  |
| **Labs – Biomarkers** | | | | | |
| **D-dimer** | |  |  |  | 0.844 |
|  | Median | 2.5 | 2.4 | 2.4 |  |
|  | Q1, Q3 | 1.2, 5.6 | 1.4, 6.9 | 1.4, 6.7 |  |
| **Ferritin** | |  |  |  | 0.9 |
|  | Median | 962 | 1013 | 1013 |  |
|  | Q1, Q3 | 456.0, 2033.8 | 473.0, 2218.0 | 468.5, 2197.5 |  |
| **C-reactive protein** | |  |  |  | 0.655 |
|  | Median | 135.6 | 119 | 121 |  |
|  | Q1, Q3 | 78.2, 176.5 | 59.0, 184.0 | 64.5, 177.5 |  |
| **High-sensitivity troponin** | |  |  |  | 0.222 |
|  | Median | 25.5 | 33 | 33 |  |
|  | Q1, Q3 | 13.8, 60.5 | 17.5, 80.0 | 16.0, 78.5 |  |
| **Interleukin-6** | |  |  |  | 0.72 |
|  | Median | 59.9 | 50.4 | 51.5 |  |
|  | Q1, Q3 | 31.7, 87.4 | 24.2, 91.7 | 25.1, 90.4 |  |
| **Lactate dehydrogenase** | |  |  |  | 0.383 |
|  | Median | 522.5 | 572 | 548 |  |
|  | Q1, Q3 | 435.5, 610.5 | 420.5, 663.1 | 420.5, 658.5 |  |
| **Creatinine kinase** | |  |  |  | 0.347 |
|  | Median | 370 | 272 | 282 |  |
|  | Q1, Q3 | 128.8, 794.5 | 132.0, 634.5 | 129.5, 729.5 |  |
| **Prothrombin time** | |  |  |  | 0.153 |
|  | Median | 14.7 | 15.2 | 14.9 |  |
|  | Q1, Q3 | 13.7, 16.1 | 14.1, 17.2 | 13.9, 17.1 |  |
| **Inpatient Treatment** | | | | | |
| **Remdesivir** | | 6 (21.4%) | 36 (37.9%) | 42 (34.1%) | 0.112 |

*ED*, emergency department*; HFNC*, high-flow nasal cannula; *SOFA,* sequential organ failure assessment; *ICU*, intensive care unit; *F*, Fahrenheit; *RBC*, red blood cell.
