# Supplementary material for: Emergency Department-initiated High-flow Nasal Cannula for COVID-19 Respiratory Distress
Source: West J Emerg Med. 2021 Jul 20;22(4):979–87. doi: 10.5811/westjem.2021.3.50116 (PMC8328178; doi:10.5811/westjem.2021.3.50116)
Supplement: Supplementary file 2 [file wjem-22-979-s002.docx]

**Supplemental Methods**

Timing of Respiratory Events

The timing of respiratory interventions was determined by grouping respiratory flowsheet events by patient ID and oxygen delivery method. The earliest timestamps for HFNC and intubation during each encounter were saved for each patient where applicable. The time interval between each HFNC and ETT event was calculated relative to the maximum emergency department ADT time. ED departure to intervention intervals with negative values were coded as having occurred in the emergency department and intervals with positive values were coded as having occurred after ED departure. The maximum level of respiratory support for each patient was calculated at the time of ED departure, as well as at any time within the first 24 hours of hospitalization. Patients who transiently received HFNC prior to intubation were coded as requiring intubation given that this was their maximal requirement.

Vital Signs

Vital signs for each patient were extracted from the flowsheet table using the maximum values for temperature, heart rate, and respiratory rate; and the minimum values for pulse oximetry and systolic blood pressure at any time prior to ED departure. Triage and nursing notes that contained “%”, “sat”, or “SpO2” were also flagged and reviewed since many of the worst SpO2 values were only recorded in notes and not recorded in the flow sheet.

P/F Ratio

All PaO2 results prior to ED departure and during the first 24 hours of hospitalization were extracted along with the specimen collection time. Similarly, all FiO2 flowsheet values and entry timestamps were extracted for the same period. After joining these two datasets, the time between each PaO2 specimen collection time and each FiO2 entry time was calculated, all values less than zero were removed (PaO2 was collected prior to FiO2 value) and the rows with the minimum time interval between PaO2 and FiO2 were retained for each PaO2 result. The mean PaO2 result for each patient encounter was then calculated and used for subsequent analysis.

Laboratory Values

The laboratory values identified as being the earliest ordered, resulted, and/or collected by timestamps during each patient encounter were also extracted. Lab values containing error messages were excluded and any lab value below or above a laboratory defined cutoff was set to the value of the cutoff.

Body Mass Index
Patient BMI was extracted using the median value during the study period; in the four instances where this value was not directly available, BMI was calculated using the median weight and median height during the study period.
